# Bystander intervention among secondary school pupils: Testing an augmented Prototype Willingness Model

**DOI:** 10.1111/bjso.12534

**Published:** 2022-03-23

**Authors:** Stefania Pagani, Simon C. Hunter, Mark A. Elliott

**Affiliations:** ^1^ University of Strathclyde Glasgow UK; ^2^ Glasgow Caledonian University Glasgow UK; ^3^ University of Western Australia Crawley WA Australia

**Keywords:** bystander intervention, decision‐making, gender‐based violence, prototype willingness model, theory of planned behaviour

## Abstract

This study augmented the Prototype Willingness Model (PWM) to assess reactive and deliberative decision‐making underpinning bystander intervention in gender‐based violence contexts. There were 2079 participants (50% male, 49% female, and 1% unreported), aged 11–15 years old (*M* = 12.32, *SD* = 0.91), attending 19 secondary schools across Scotland. Participants self‐reported the augmented PWM variables, then their intervention behaviour approximately 1 month later. Path analyses mostly supported the predicted relationships between positive and negative bidimensional attitudes, subjective norms, prototype perceptions, perceived behavioural control, and self‐efficacy on intentions and willingness. Willingness predicted positive (speaking with a teacher) and negative (doing nothing) intervention in less serious violence. Self‐efficacy predicted negative intervention in more serious violence. Subjective norms positively moderated the attitudes–intentions relationship. Overall, the results suggested that reactive (willingness) more so than deliberative (intention) decision‐making account for intervention when young people witness gender‐based violence. Additionally, the findings highlight the complexity of bystander intervention decision‐making, where adding control perceptions, bidimensional attitudes, and moderators have independent contributions. Furthermore, self‐comparison to the typical bystander who positively intervenes (prototype perceptions) was the strongest predictor of intentions and willingness, highlighting in a novel way the importance of image and group membership on decision‐making.

## INTRODUCTION

Gender‐based violence is aggression targeted at someone because of the gender with which they identify. This can include verbal, emotional, physical, and sexual aggression (Björkqvist, 1994; Wang et al., 2009). It is one of the most common forms of violence in the United Kingdom and tends to be targeted towards women and girls (Batchelor et al., 2019; ONS, 2019, 2021; Scottish Government, 2018; Stark, 2007). Regarding sexual assault specifically, in the year ending March 2020, there were 773,000 victims aged 16–74 years in England and Wales, with almost four times as many female victims as male victims (ONS, 2021). Violence among young people in schools usually occurs in front of others (Hawkins et al., 2001; Katz, 1995; O'Connell et al., 1999; Pepler & Craig, 1995; Polanin et al., 2012; Salmivalli, 2014). The presence of bystanders in violent situations puts them in a position where they can influence the outcome (Katz et al., 2011; Polanin et al., 2012). Furthering the understanding of bystander intervention in gender‐based violence contexts can therefore support reduction efforts. Bystander intervention involves a level of planning (McMahon et al., 2015; Rosval, 2013) but the potentially reactive nature of intervention remains unexplored. Self‐efficacy is also clearly important in explaining intervention (Sjögren et al., 2020; Sundstrom et al., 2018), however, there is a need to differentiate this from other control factors. Furthermore, moderation contributions between social cognitive variables have not been examined in the context of bystander intervention. This study addresses these issues by testing the predictive ability of an augmented version of the Prototype Willingness Model (PWM: Gibbons & Gerrard, 1995, 1997).

The PWM is aligned to dual‐process theories of decision‐making (Chaiken & Trope, 1999), which consider both deliberative and reactive processes. The deliberative pathway is governed by *intentions* that are antecedent to behaviour. Importantly, intentions are goal‐driven and reflect conscious planning. The reactive pathway is governed by *willingness*, also antecedent to behaviour. This pathway accounts for an individual's general openness to perform behaviours and requires little deliberation. Willingness and intentions directly predict behaviour and are both predicted by *attitudes* (positive and/or negative evaluations) and *subjective norms* (perceptions of others’ behaviours). Willingness is also predicted by *prototype perceptions*. Prototypes are highly accessible images of the typical person who performs a behaviour that is drawn from memory without much thought. As a result, it is expected that prototype perceptions (perceived similarity to the prototype) will predict willingness but not intentions in the PWM (Gibbons & Gerrard, 1995).

The PWM was originally conceptualized to explain health‐risk behaviours involving reaction, and little planning (Gibbons & Gerrard, 1995, 1997). There are numerous studies within the literature supporting its applicability to these behaviour contexts. However, there is also evidence for its predictive ability in health‐protective behaviour contexts, such as exercise (for meta‐analyses see Todd et al., 2016, and van Lettow et al., 2016). The PWM has also been particularly important in explaining young people's behaviours (Gibbons & Gerrard, 1995, 1997). Adolescence is a period in which there is much developmental and relationship change, where gender roles are explored and interpersonal relationships are negotiated (Basow & Rubin, 1999; Katz et al., 2011). There is therefore a need to test the PWM in the context of bystander intervention in gender‐based violence situations among adolescents.

### PWM components

**FIGURE 1:**
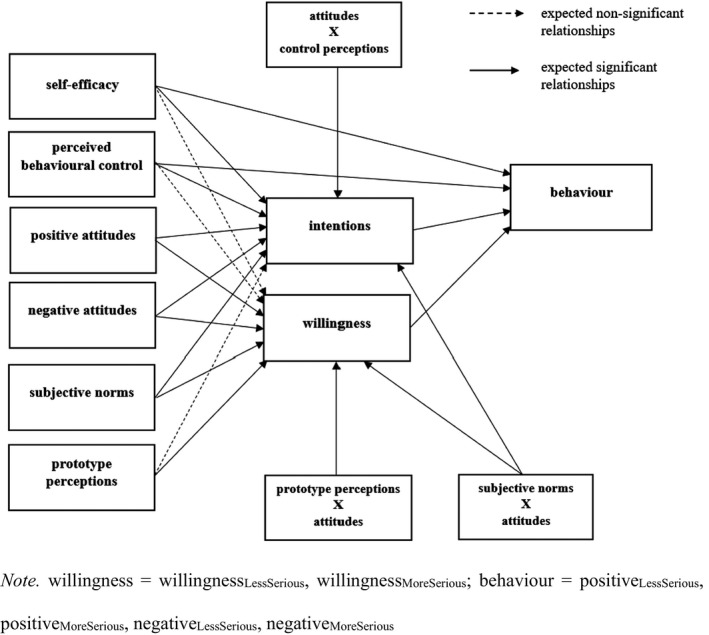
Full augmented Prototype Willingness Model

This study was conducted to test an augmented PWM in the context of bystander intervention in gender‐based violence. The augmented model (Figure 1) comprized both positive and negative attitudes as potential independent predictors of intentions and willingness. Although attitudes predict both intentions and willingness in different contexts (Elliott et al., 2017; Rivis & Sheeran, 2003; Rivis et al., 2006), attitudes have not always aligned with behaviours in the bystander and bullying literature (Rigby & Slee, 1991; Rosval, 2013; Salmivalli, 2014; Salmivalli et al., 2005; Salmivalli & Voetan, 2004). For example, bystanders may not intervene despite displaying positive attitudes towards the victim and negative attitudes towards the perpetrator and violence itself (Ortega & Mora‐Merchan, 1999). One explanation is that participants’ attitudes towards specific people (the victim or perpetrator of the bullying) have been used to predict behaviour (intervening to stop violence) and attitudes towards behaviour (the act of intervening) are needed for more exacting behavioural prediction (Ajzen, 1991; Fishbein & Ajzen, 1975).

However, attitudes towards behaviours are not always strong predictors of intentions to intervene (see Rosval, 2013). Historically, attitudes have been measured as unidimensional constructs (ranging from positive to negative; Osgood et al., 1957). Indeed, within the PWM attitudes are conceptualized as unidimensional (Gibbons & Gerrard, 1995). However, recently, attitudes have been considered as bidimensional (both positive and negative) constructs (Elliott et al., 2015; McCartan et al., 2018). This bidimensional approach may be more appropriate for bystander intervention since it is characterized as having equally salient positive and negative outcomes (Debnam & Mauer, 2021; Hoxmeier et al., 2018; McLaughlin et al., 2005; Rigby & Johnson, 2005; Thornberg et al., 2012, 2018). For example, intervention can be characterized by positive outcomes with an external focus on others (*“if I intervene I will help the victim”*), and negative outcomes with an internal focus on the bystander (*“if I intervene the perpetrator might turn on me”*). This study will therefore take a bidimensional approach to separately measure positive and negative attitudes, and test whether they can independently predict bystander intervention.

Subjective norms and prototype perceptions are important social influences in decision‐making (Armitage & Conner, 2000, 2001; Chen et al., 2016; Elliott et al., 2017; Manning, 2009; Rivis & Sheeran, 2003; Wilson et al., 2016). Subjective norms account for 85% of the variance in intentions to intervene in bullying situations (Rosval, 2013). Prototype perceptions are likely to be important for adolescents as they involve comparing oneself with others, and image and group membership are important components of adolescents’ social lives (Levine et al., 2002; Levine & Crowther, 2008; Lloyd & Lucas, 1998; Palmer & Abbott, 2018; Rivis et al., 2006; Rutland & Killen, 2015, 2017; Salmivalli, 2010, 2014). Though they have not previously been measured in a bystander intervention context, it is expected that young people will be more willing to intervene in gender‐based violence if they identify with the ‘typical’ bystander.

Behavioural intentions can be conceptualized as planning to perform a behaviour, as well as the perceived likelihood of doing so (Armitage et al., 2015; Gibbons et al., 1998). Within the bystander literature, the predictive ability of intentions on intervention has yielded promising results (DeSmet et al., 2016; McMahon et al., 2015; Rosval, 2013). Similarly, behavioural willingness involves a person's openness and motivation to perform a behaviour. Theoretically, willingness is believed to increase when someone encounters facilitating situations (Gibbons & Gerrard, 1995, 1997). Willingness, as conceptualized in the PWM, has not been examined in the context of bystander intervention, however, is likely to be important because violence in schools is dynamic and likely to involve situation‐specific motivation.

One assumption of the PWM is that behaviours are mostly volitional but often not intentional (Gerrard et al., 2008). Other models, such as the Theory of Planned Behaviour (TPB; Ajzen, 1988, 1991), stress the importance of volition in decision‐making (Ajzen, 2002) and perceived behavioural control serves as a proxy to actual control. Perceived behavioural control predicts behaviour either directly or indirectly through intentions. This involves a deliberative process in which external opportunities or resources are considered which influence behaviour performance (Fishbein & Ajzen, 2010; Terry & O’Leary, 1995). Despite being absent in the PWM, perceived behavioural control can account for 79% of the variance in intentions (Rosval, 2013). There is also evidence showing that bystanders who intervene positively have greater levels of perceived behavioural control (Hoxmeier et al., 2018).

There is therefore a strong case for including perceived behavioural control in models of bystander intervention. In addition, self‐efficacy is important to consider as it is theoretically distinct (Ajzen, 2002), reflecting an individual's internal beliefs in their capabilities to perform a behaviour (Bandura, 1977, 1997). Self‐efficacy involves perceived skill and competency over a behaviour (Ajzen, 1991). Existing studies tend to replace or conflate perceived behavioural control with self‐efficacy (Banyard, 2008; Banyard & Moynihan, 2011; Coker et al., 2011; McMahon et al., 2015; Pöyhönen et al., 2010; Rosval, 2013; Salmivalli, 2010; Sjögren et al., 2020; Sundstrom et al., 2018). However, these factors are likely to provide independent insight into intervention as factor analytic studies illustrate their independent effects on intentions and behaviour (Armitage & Conner, 1999a, 1999b; Conner & Armitage, 1998; Manstead & van Eekelen, 1998; Sparks et al., 1997; Terry & O’Leary, 1995). Since control perceptions govern the deliberative pathway, they are expected to predict intentions but not willingness.

Decision‐making is a complex process where explanatory factors will interact and so our augmented PWM reflects this. For example, social influences (subjective norms) moderate the relationship between attitudes and intentions in other behaviour contexts (Conner & McMillan, 1999; Schüz et al., 2019). This aligns with the contingent‐consistency hypothesis (Acock & DeFleur, 1972) which posits that attitudes will predict decisions to act when the social environment supports that behaviour. Additionally, attitudes can moderate the relationship between control perceptions and intentions (Eagly & Chaiken, 1993). This moderation effect has been demonstrated in other contexts (Conner & McMillan, 1999; Wolff et al., 2011; Yardley et al., 2007). This study is the first to examine interactions between TPB and PWM social‐cognitive factors in the context of bystander decision‐making by examining the moderating contributions of (i) social influences on attitudes and (ii) attitudes on control perceptions.

### Present study and hypotheses

This study utilizes the PWM to understand the individual reactive and deliberative decision‐making processes underpinning adolescents’ intervention in gender‐based violence contexts. Additionally, an augmented version of the PWM will be examined in which bidimensional attitudes, control perceptions, and moderators are included (see Figure 1).

As pre‐registered on the Open Science Framework (OSF: [blinded for peer review]), it was hypothesized that: (H1a) Attitudes and subjective norms will account for a significant portion of the variance in intentions and willingness. At the same time, prototype perceptions will account for a significant portion of the variance in willingness, not intentions. Furthermore perceived behavioural control and self‐efficacy will account for a significant portion of the variance in intentions, not willingness. (H1b) Subsequently, willingness and intentions will account for a significant portion of the variance in intervention. (H2) Perceived behavioural control and self‐efficacy will account for a significant portion of variance in intervention. (H3) Intentions will mediate the relationship between the predictors (attitudes and subjective norms) and intervention. At the same time, willingness will mediate the relationship between the predictors (attitudes, subjective norms, and prototype perceptions) and intervention. (H4) Positive attitudes will be better predictors of intentions and willingness when subjective norms and prototype perceptions are high versus low, and low versus high for negative attitudes. Perceived behavioural control and self‐efficacy will be better predictors of intentions when positive attitudes are high versus low and negative attitudes are low versus high. For each hypothesis, positive relationships are expected, except for those relating to negative attitudes and negative intervention, where negative relationships are expected.

## METHOD

### Participants

A total of 2079 young people attending the first 3 years of schooling (S1–S3) in 19 mainstream secondary schools in Scotland participated between fall 2018 and spring 2019. Participants were aged 11–15 years old^1^ (*M* = 12.32, *SD* = 0.91), of whom 1011 (48.70%) reported their gender as female, 1040 (50.02%) as male, 17 (0.80%) as “prefer not to say,” and 10 (0.50%) were missing. The sample predominantly identified as “White Scottish or White British” (*N* = 1876; 90.30%), 3.10% as “Asian, Asian Scottish/ British,” 0.80% as “African,” 1.70% as “Mixed or multiple ethnic group,” 0.50% as “Caribbean or Black,” and 3.00% as “Other ethnic group.” The percentage of pupils registered for free school meals in each school was used as a proxy for socioeconomic status and ranged from 1.00% to 50.20% (*M* = 20.60%, *SD* = 14.00). Across schools, age ranged from *M* = 11.67 to 13.77; percentages of females ranged from 42.86% to 78.57%; and percentages of “White Scottish or White British” participants ranged from 57.14% to 97.33%.

The sample size exceeded the pre‐registered target sample size (*N* = 1146; [blinded for peer review]). However, the research team aimed to over recruit participants due to the likelihood that not all participants would report witnessing more and less serious violence, resulting in smaller subsets in the analyses (see Pre‐analytic work). Furthermore, as noted in the pre‐registration, multicollinearity was anticipated and so separate analyses were planned for each of the moderators. When including one moderator, G*Power indicated a required sample of 652 participants.

### Measures

All measures used in the study are available on OSF (blinded for peer review). A prospective correlational design was used, with two‐time points. Time 1 measured predictors and mediators (attitudes, control, social influences, intentions, and willingness). Time 2 (1‐month later) measured outcomes (positive and negative intervention).

#### *Gender*‐*based violence*

Eight gender‐based violence examples developed by Miller et al. (2012) were employed across measures. These included examples of boys being violent towards girls. Scale items were drawn from the literature (Elliott et al., 2015, 2017; Miller et al., 2012; Thornberg & Jungert, 2014; Wilson et al., 2016), and incorporated examples of emotional, verbal, physical, and sexual violence, such as: “A male peer/friend spreading rumours about a girl's sexual reputation, like saying she's ‘easy to get with’…” This approach reflected calls to include a range of situations (Katz et al., 2011; Miller et al., 2012). All eight examples of gender‐based violence were not used for every measure to reduce the burden on participants. However, examples were included on the basis that a balanced selection of verbal/emotional and physical/sexual violence items assessed each construct, and all participants were exposed equally to each of the eight examples. Factor analyses were conducted on all measures (see Results). Subsequently, factor scores were generated for each measure by multiplying each raw score by its factor weight and summing those results. These scores were used in all analyses.

### T1 measures

#### Attitudes

Two six‐item measures (Elliott et al., 2015) were adapted, one to assess positive attitudes (“positive,” “beneficial,” “rewarding,” “pleasant,” “enjoyable,” and “advantageous”) and one negative attitudes (“negative,” “harmful,” “unrewarding,” “unpleasant,” “unenjoyable,” and “disadvantageous”). Pupils were asked to rate their responses to questions like, “How positive would it be if you did something about it when you saw… (*violence example*),” from “not at all positive” = 1 to “extremely positive” = 9, and “How negative would it be if you did something about it when you saw… (*violence example*),” from “not at all negative” = 1 to “extremely negative” = 9. Higher factor scores indicated either more positive or more negative attitudes. Internal reliability was satisfactory (positive scale α = .93; negative scale α = .93).

#### Subjective norms

Wilson et al.’s (2016) three‐item scale was adapted by asking participants to rate their response to questions like, “Of the students you know, how many do you think will do something about it over the next month when they see… (*violence example*),” from “none of them” = 1 to “all of them” = 9. Higher factor scores indicated higher subjective norms. Internal reliability was satisfactory (α = .82).

#### Perceived behavioural control

Wilson et al.’s (2016) two‐item scale was adapted by asking participants to rate their response to questions like, “Over the next month, how much personal control do you feel you have over doing something about it when you see… (*violence example*),” from “no control at all” = 1 to “complete control” = 9. Higher factor scores indicated high perceived behavioural control. Internal reliability was satisfactory (α = .65).

#### Self‐efficacy

Wilson et al.’s (2016) three‐item scale was adapted by asking participants to rate their responses to questions like “Over the next month, I have the ability to do something about it when I see… (*violence example*),” from “not at all confident” = 1 to “very confident” = 9. Higher factor scores indicated high self‐efficacy. Internal reliability was satisfactory (α = .75).

#### Prototype perceptions

Elliott et al.’s (2017) four‐item scale was adapted by asking participants to rate their responses to questions like “Do you resemble the type of person your age that regularly does something about it when they see… (*violence example*),” from “definitely no” = 1 to “definitely yes” = 9. Higher factor scores indicated high prototype perceptions. Internal reliability was satisfactory (α = .91).

#### Intentions

Miller et al.’s (2012) eight‐item scale was adapted by asking participants to rate their responses to questions like “How likely are you to do something about it over the next month if a male peer/friend is… (*violence example*),” from “very unlikely” = 1 to “very likely” = 5. Higher factor scores indicated higher intentions. Internal reliability was satisfactory (α = .95).

#### Willingness

Elliott et al.’s (2017) three‐item scale was adapted. Given that willingness involves situation‐specific determinants of behaviour, social influences were controlled to allow for the examination of individual factors (Ajzen, 2011). Therefore, three specific social circumstances influence intervention similarly (Bennett et al., 2014; Latané & Darley, 1968; Levine & Crowther, 2008; Palmer & Abbott, 2018; Salmivalli, 2010) were included. Participants were asked to rate their responses to questions like, “Suppose you saw… (*violence example*)…over the next month and… no‐one else there was doing anything about it/ none of your friends were intervening/ no‐one else was around. How willing would you be to do something?” From “not at all willing” = 1 to “extremely willing” = 9. Two factors were identified (relating to more and to less serious violence examples), and higher factor scores indicated higher willingness. Internal reliability was satisfactory (willingness_MoreSerious_: α = .82; willingness_LessSerious_: α = .86).

### Covariates (see Appendix S1 for relevant correlations)

#### Gender

Participants reported their gender as “a boy,” “a girl,” or “prefer not to say.” Subsequently, those who responded as “prefer not to say,” or whose responses were missing were deleted from analyses due to small numbers (see participants section). Gender was coded “boy = 0” and “girl = 1.”

#### Age

Participants reported their age from 11 to 15. No treatment of this variable occurred for analyses.

#### Ethnicity

Participants reported their ethnicity as “White Scottish or White British,” “Asian, Asian Scottish/British,” “African,” “Mixed or multiple ethnic group,” “Caribbean or Black,” and “Other ethnic group.” Due to small numbers in the responses from those who did not identify as “White Scottish or White British” (see Participants section), all other ethnic groups were collapsed into one group “other ethnic group.” Therefore, ethnicity was coded as “0 = White Scottish or White British,” and “1 = other ethnic groups” for analyses.

#### Moral disengagement

Thornberg and Jungert’s (2014) six‐item scale was adapted by asking participants responses to questions like, “It's okay for a male peer to shove, grab, or otherwise physically hurt a girl who they don't like,” from “strongly agree” = 1 to “strongly disagree” = 7. Higher factor scores meant higher moral disengagement. Internal reliability was satisfactory (α = .91).

#### Empathy

The six‐item scale from Caravita et al. (2009) was used. Gender‐based violence situations were not incorporated into this scale as it measures a personality trait (Barkoukis et al., 2016; Caravita et al., 2009; Miller & Eisenberg, 1988). In this measure, participants were asked to rate their responses to questions like, “Seeing a friend crying makes me feel as if I am crying,” from “never true” = 1 to “always true” = 4. Mean scores were generated where a higher score indicated higher affective empathy. Internal reliability was satisfactory (α = .77).

### **T2** **measures**

#### *Self*‐*reported intervention*

The same eight gender‐based violence examples were used to assess actual intervention opportunities and behaviours. Each participant could therefore report they had witnessed between 0 and 8 gender‐based violence situations in the preceding month (median = 3.00, range = 0–8). For each situation they had witnessed, participants reported how they had intervened by ticking a box, or boxes, aligning with Miller et al.’s (2012) two negative (e.g., “*I didn't do*/*say anything*”) and four positive (e.g., “*I told the person in public that acting like that was not ok*”) (see Appendix S2) intervention possibilities. Two proportion scores were calculated for every participant, one for positive and one for negative intervention responses. These scores represented the proportion of times participants reported intervening given the number of opportunities they had to intervene.

The distribution of data for both outcome behaviours was trimodal. Therefore, the proportion scores were divided into three categories. Participants who reported never intervening positively were coded 0; those who intervened positively some of the time as 1; and those who intervened positively all the time as 2 (resulting in 54.15%, 18.10%, and 27.75%, respectively). The same method was applied to negative intervention (resulting in 28.35%, 19.20%, and 52.45%, respectively).

### Procedure

Ethical approval was granted by the lead author's institution. Local Education Authorities (LEAs) were contacted for permission to approach schools. Permission was obtained from 10 of 13 (77%) LEAs. Of the schools contacted, 42 (26%) expressed interest in participating though not all were included in the study. Information letters and consent forms were distributed to parents. At the preference of the LEA, either negative (80%; 1778 participants) or positive (20%; 301 participants) consent was sought. Parents were given at least 1 week to return consent in either case. Once participants had consented, they completed the anonymous questionnaires. This was done within a classroom or assembly hall for both T1 (45–55 min) and T2 (5–10 min), with teachers and members of the research team available to answer questions. Participants were subsequently debriefed. Copies of all relevant documentation in this section are on the OSF (blinded for peer review).

## RESULTS

### Factor analyses

Two‐factor analyses were conducted on each measure (see Appendix S3). The dataset was halved randomly to achieve this. It was intended that exploratory then confirmatory analyses would be performed (e.g., Houghton et al., 2013) but this was not possible for some measures (positive attitudes, negative attitudes, perceived behavioural control, self‐efficacy, and intentions) due to problems with reverse‐scored items with poor factor loadings (<.32, Tabachnick & Fidell, 2007; see also Lindwall et al., 2012, and Weijters & Baumgartner, 2012), and issues regarding correlating similarly measured items (Byrne, 2013). Therefore, a multitrait–multimethod approach (a confirmatory factor analytic approach, where different models are tested that include correlations between similar traits and methods of measurement; see De Lima & De Souza, 2019, and Tomas & Oliver, 1999) was used for attitudes and intentions. Additionally, a theoretically informed approach was used for perceived behavioural control and self‐efficacy, where they were included in the same model representing “control,” but still retained as distinct factors (Ajzen, 2002). This also involved parcelling the reverse‐scored items with the highest loading non‐reverse‐scored items for both measures (see Appendix S3; Little et al., 1999, 2002).

Subsequently, the best fitting models were subjected to confirmatory factor analyses on the second half of the dataset (see Table 1). Most measures had two‐factor structures with emotional/verbal violence items loading onto one factor, and physical/sexual violence items loading onto the other. For all measures, except the willingness factors (*r* = .87), the two factors were correlated *r* > .90. Therefore, where the one‐factor structure also fitted the data well, this was selected as the preferred fit. The only exception was the willingness measure, where only a two‐factor structure yielded a good fit to the data. From here forward, the willingness measures are referred to as willingness_LessSerious_ and willingness_MoreSerious_.

**TABLE 1:** Results illustrating fit of each of the models to the relevant datasets

Factor analyses	df	RMSEA (90% CI)	CFI	Omega (ω)
Positive attitudes				
FA1	48	.058 (.050–.067)	.92	.90
FA2	48	.065 (.057–.074)	.90	.91
Negative attitudes				
FA1	48	.048 (.039–.057)	.94	.91
FA2	48	.046 (.037–.055)	.94	.90
PBC				
FA1	4	.042 (.009–.074)	.99	.90
FA2	4	.000 (.000–.025)	1.00	.90
Self‐efficacy				
FA1	4	.042 (.009–.074)	.99	.56
FA2	4	.000 (.000–.025)	1.00	.53
Subjective norms				
FA1	1	.034 (.000–.099)	.99	.73
FA2	1	.000 (.000–.053)	1.00	.77
Prototype perceptions				
FA1	6	.047 (.023–.074)	.98	.82
FA2	6	.053 (.028–.079)	.98	.85
Intentions				
FA1	11	.069 (.052–.087)	.94	.86
FA2	11	.074 (.057–.092)	.94	.97
Willingness				
FA1	5	.040 (.010–.070)	.99	.83
FA2	5	.089 (.064–.117)	.95	.84

Note

CFI, comparative fit index; CI, confidence interval; df, degrees of freedom; FA1, factor analysis on first half of dataset; FA2, factor analysis on second half of dataset; RMSEA, root mean square error of approximation fit index.

Correlations between the four intervention indicators’ residuals could not be considered as the model would be overfit (Babyak, 2004), leading to a poor fitting model because the indicators were measured the same way (Tomas & Oliver, 1999). When the correlations for positive_MoreSerious_ and positive_LessSerious,_ and those for negative_MoreSerious_ and negative_LessSerious_ were examined, the correlations were medium‐to‐high (*r* = .56 and *r* = .53, respectively), suggesting that more and less serious items had some distinction (Evans, 1996; Hair, 1998; Tabachnick & Fidell, 2007). Therefore, four outcomes were utilized throughout the analyses.

### Correlations

Table 2 shows the means, standard deviations, and correlations. All correlations between the predictors and the mediators (intentions and willingness) except one (negative attitudes with subjective norms) were significant, ranging from small to large (*r*s = .11 to .87). The mediators, self‐efficacy, and perceived behavioural control were significantly correlated with both positive_LessSerious_ and negative_LessSerious_ intervention, with small correlations (*r*s = .08 to .15). Perceived behavioural control and negative_LessSerious_ were not correlated. Only self‐efficacy and willingness_MoreSerious_ had significant but small correlations with both positive_MoreSerious_ (*r*_s_ = .10, .08) and negative_MoreSerious (_ *r*_s_ = −.13, −.09) intervention, respectively.

**TABLE 2:** Means (*M*), standard deviations (*SD*), and correlations between predictors, mediators, and outcome variables

	*M* (*SD*)	2	3	4	5	6	7	8	9	10	11	12	13
1. Positive_LessSerious_	.73 (.84)	.56**	−.69**	−.35**	.09*	.15**	.11**	.08*	.15**	.01	.01	.11**	.13**
2. Positive_MoreSerious_	.74 (.89)	‐	−.38**	−.63**	.03	.12**	.08*	.04	.10*	.03	−.01	.07	.11**
3. Negative_LessSerious_	.70 (.29)		‐	.53**	−.09**	−.15**	−.10**	−.06	−.13**	−.04	.01	−.08*	−.12**
4. Negative_MoreSerious_	.81 (.26)			‐	−.06	−.14**	−.09*	−.02	−.13**	−.05	.03	−.08*	−.13**
5. Intentions	2.64 (.96)				‐	.45**	.41**	.22**	.35**	.33**	−.24**	.22**	.38**
6. Willingness_LessSerious_	2.93 (.97)					‐	.87**	.40**	.56**	35**	−.12**	.40**	.53**
7. Willingness_MoreSerious_	3.10 (.95)						‐	.37**	.52**	.35**	−.11**	.38**	.53**
8. PBC	2.96 (.90)							‐	.56**	.21**	−.15**	.23**	.32**
9. Self‐efficacy	1.85 (.56)								‐	.27**	−.18**	.38**	.48**
10. Positive attitudes	3.00 (1.36)									‐	−.51**	.27**	.33**
11. Negative attitudes	2.80 (1.55)										‐	−.05	−.11**
12. Subjective norms	2.44 (.87)											‐	.38**
13. Prototype perceptions	4.22 (1.62)												‐

Correlations for variables 1–4 are Spearman's Rho (*r_s_*); correlations for variables 5–13 are Pearson's (*r*).

Abbreviation: PBC, perceived behavioural control.

*p < .05, **p < .01.

### Pre‐analytic work

All interaction terms had VIF scores exceeding 10 (Hair et al., 1998), identifying multicollinearity as problematic. Therefore, factor scores were mean‐centred (Kraemer & Blasey, 2004) for predictor variables before creating interaction terms.

In the pre‐registration, we planned to conduct one linear regression path analysis which incorporated all interaction terms, using Mplus Version 7.31 (Muthén & Muthén, 1998–2012). Mplus allows missing data to be addressed using Full Information Maximum Likelihood. However, this approach was changed in three key ways. First, the trimodal distribution of the outcome variables required the use of logistic regression with Monte Carlo integration. Second, we conducted the analysis over two separate steps instead of one. That is, in step 1 the PWM factors were examined, and in step 2 the additional factors (perceived behavioural control and self‐efficacy) were also examined. This further analytical step was required to draw more concrete conclusions regarding the need to augment the PWM. Third, as multicollinearity was an issue, separate models were estimated for each of the interaction terms.

Furthermore, the model was split into two: (i) for the outcomes positive_LessSerious_ and negative_LessSerious_ and (ii) for the outcomes positive_MoreSerious_ and negative_MoreSerious_. This addressed statistical power since only including participants who had the opportunity to intervene in *both* more and less serious violence reduced the analytical sample size to 617 participants. Estimating two models resulted in larger analytic samples (*N* = 711 in the “more serious” and *N* = 1036 in the “less serious” model). In addition, positive and negative interventions were assessed together in two models (one for positive and negative intervention in less serious episodes of gender‐based violence, and one for more serious episodes) instead of four because of evidence that they are associated (Thornberg & Wänström, 2018). Indeed, both pairs of outcomes were highly, negatively correlated (positive_MoreSerious_ with negative_MoreSerious_, *r* = −.63; positive_LessSerious_ with negative_LessSerious_, *r* = −.69).

Overall, there were sixteen path analyses conducted: two (*N* = 711 for the “more serious” model, *N* = 1036 for the “less serious” model) for the first step, and two for the second step for the main models without the interactions, and twelve for the main models with the interactions (*N* = 1036). Gender, age, ethnicity, moral disengagement, and affective empathy were covariates due to their impact on bystander intervention (Brown et al., 2014; Burns et al., 2019; Caravita et al., 2009; Jenkins et al., 2018; Menolascino & Jenkins, 2018; Thornberg & Jungert, 2014; Thornberg et al., 2012).

### Path analyses

#### Hypothesis 1a

Step 1 revealed that the models accounted for medium‐large (Cohen, 1992) variance in intentions and willingness (Table 3). Regarding the deliberative pathway, positive attitudes positively, and negative attitudes negatively predicted intentions. However, disconfirming our hypothesis, prototype perceptions positively predicted, and subjective norms did not predict intentions. Regarding the reactive pathway, positive attitudes, subjective norms, and prototype perceptions positively predicted both willingness measures. However, negative attitudes did not predict the willingness measures.

**TABLE 3:** Effect sizes, standardized estimates, and *p*‐values for predictors on mediators

Step 1	Intentions		Willingness_LessSerious_		Willingness_MoreSerious_	
Effect sizes	*R* ^2^ = .20***		*R* ^2^ = .35***		*R* ^2^ = .31***	
	*β*	*p*‐value	*β*	*p*‐value	*β*	*p*‐value
Positive attitudes	.11	.014	.18	.000	.17	.000
Negative attitudes	−.15	.000	.04	.318	.01	.894
Subjective norms	.04	.209	.20	.000	.14	.003
Prototype perceptions	.32	.000	.42	.000	.41	.000

Step 2	Intentions		Willingness_LessSerious_		Willingness_MoreSerious_	
Effect sizes	*R* ^2^ = .22***		*R* ^2^ = .44***		*R* ^2^ = .37***	
	*β*	*p*‐value	*β*	*p*‐value	*β*	*p*‐value
PBC	.00	.954	.06	.048	.10	.021
Self‐efficacy	.18	.000	.31	.000	.42	.000
Positive attitudes	.10	.018	.18	.000	.12	.000
Negative attitudes	−.13	.001	.08	.058	.03	.314
Subjective norms	.01	.877	.13	.000	.08	.092
Prototype perceptions	.26	.000	.30	.000	.18	.000

Note

Abbreviation: PBC, perceived behavioural control.

***p < .001.

Step 2 revealed that the models accounted for more variance in intentions and the willingness measures (Table 3) when perceived behavioural control and self‐efficacy were added. Furthermore, these increases were all significant (see Appendix S4). Regarding the deliberative pathway, the results were the same as in step 1. Additionally, self‐efficacy positively predicted intentions but disconfirming our hypothesis perceived behavioural control did not. Regarding the reactive pathway, the results were the same as in step 1. Disconfirming our hypothesis, perceived behavioural control and self‐efficacy also positively predicted both willingness measures.

#### Hypotheses 1b and 2

Step 1 revealed that the models accounted for significant variance in both positive_LessSerious_ and negative_LessSerious_ interventions (Table 4). Willingness_LessSerious_ positively predicted positive_LessSerious_ where, with each unit increase in willingness, participants were 1.25 times more likely to intervene positively. Additionally, willingness_LessSerious_ negatively predicted negative_LessSerious_ intervention where with each unit increase in willingness participants were .79 times less likely to intervene negatively. Intentions were not a significant predictor. Step 2 revealed when perceived behavioural control and self‐efficacy were added, the variance accounted for by the variables increased. However, there were no significant contributions on the less serious outcomes, and the contributions of willingness_LessSerious_ also became non‐significant.

**TABLE 4:** Effect sizes, unstandardized estimates (logits), log odds, and *p*‐values for predictors of bystander intervention in less serious gender‐based violence contexts

Step 1	Positive_lessSerious_			Negative_LessSerious_		
Effect sizes	*R* ^2^ = .04**			*R* ^2^ = .03*		
	*B*	*(Exp)B*	*p*‐value	*B*	*(Exp)B*	*p*‐value
Intentions	.03	1.03	.740	−.02	.98	.835
Willingness_LessSerious_	.22	1.25	.019	−.24	.79	.013
Covariates						
Age	−.18	.83	.005	1.14	.13	.032
Gender	.13	1.14	.344	.77	−.26	.058
Ethnicity	.16	1.17	.443	.78	−.25	.215
Affective empathy	.02	1.02	.864	1.03	.03	.792
Moral disengagement	−.17	.84	.038	1.04	.04	.649

Step 2	Positive_lessSerious_			Negative_LessSerious_		
Effect sizes	*R* ^2^ = .05**			*R* ^2^ = .04**		
	*B*	*(Exp)B*	*p*‐value	*B*	*(Exp)*B	*p*‐value
Intentions	.01	1.01	.950	.01	1.01	.955
Willingness_LessSerious_	.16	1.17	.142	−.18	.83	.094
PBC	.02	1.02	.817	.02	1.02	.826
Self‐efficacy	.23	1.23	.184	−.24	.79	.167
Covariates						
Age	−.18	.84	.005	.13	1.14	.034
Gender	.14	1.15	.301	−.27	.76	.051
Ethnicity	.14	1.14	.504	−.23	.79	.259
Affective empathy	.03	1.03	.799	.02	1.02	.857
Moral disengagement	−.15	.86	.064	.02	1.02	.771

Note

Abbreviation: PBC, perceived behavioural control.

*p < .05, **p < .01.

Step 1 revealed that the models accounted for significant variance in positive_MoreSerious_, and negative_MoreSerious_ intervention (Table 5). However, there were no significant contributions for willingness_MoreSerious_ or intentions. When perceived behavioural control and self‐efficacy were added in step 2, the variance accounted for by the variables increased. The increase for negative_MoreSerious_ was significant, however, that for positive_MoreSerious_ was not. There were no significant contributions for positive_MoreSerious_ intervention. However, supporting our hypothesis, self‐efficacy negatively predicted negative_MoreSerious_ intervention, where with each one‐unit increase in self‐efficacy, participants were .56 times less likely to intervene negatively. Also, disconfirming our hypothesis, perceived behavioural control positively predicted negative_MoreSerious_ intervention, where with each one‐unit increase in perceived behavioural control, participants were 1.24 times more likely to intervene negatively.

**TABLE 5:** Effect sizes, unstandardized estimates (logits), log odds, and *p*‐values for predictors of bystander intervention in more serious gender‐based violence contexts

Step 1	Positive_MoreSerious_			Negative_MoreSerious_		
Effect sizes	*R* ^2^ = .05**			*R* ^2^ = .01*		
	*B*	*(Exp)B*	*p*‐value	*B*	*(Exp)B*	*p*‐value
Intentions	−.06	.94	.554	.03	1.03	.798
Willingness_MoreSerious_	.15	1.16	.153	−.16	.86	.138
Covariates						
Age	−.21	.81	.010	.08	1.08	.309
Gender	−.03	.98	.877	.03	1.03	.839
Ethnicity	.51	1.67	.026	−.70	.50	.003
Affective empathy	.17	1.19	.216	−.21	.81	.124
Moral disengagement	−.21	.81	.028	.14	1.51	.124

Step 2	Positive_MoreSerious_			Negative_MoreSerious_		
Effect sizes	*R* ^2^ = .06**			*R* ^2^ = .06**		
	*B*	*(Exp)B*	*p*‐value	B	*(Exp)B*	*p*‐value
Intentions	−.09	.92	.398	.07	1.07	.513
Willingness_MoreSerious_	.08	1.08	.514	−.07	.94	.576
PBC	−.09	.91	.419	.21	1.24	.048
Self‐efficacy	.35	1.41	.089	−.58	.56	004
Covariates						
Age	−.21	.81	.011	.08	1.08	.325
Gender	−.02	.98	.919	.03	1.03	.867
Ethnicity	.50	1.64	.031	−.68	.51	.005
Affective empathy	.19	1.20	.190	−.24	.79	.090
Moral disengagement	−.19	.82	.047	.12	1.13	.207

Note

Abbreviation: PBC, perceived behavioural control.

*p < .05, **p < .01.

#### Hypothesis 3

Regarding less serious violence, Step 1 showed positive indirect, but trivial, contributions from the predictors, positive attitudes (*B*_1_ = .03, *p* = .029), subjective norms (*B*_1_ = .05, *p* = .035), and prototype perceptions (*B*_1_ = .06, *p* = .022) to positive_LessSerious_ through willingness_LessSerious_, where with each simultaneous one‐unit increase in the predictors and willingness_LessSerious_, participants were respectively 1.03, 1.05, and 1.06 times more likely to intervene positively. Additionally, there were significant negative indirect, but trivial, contributions for predictors, positive attitudes (*B*_1_ = −.03, *p* = .024), subjective norms (*B*_1_ = −.05, *p* = .024), and prototype perceptions (*B*_1_ = −.06, *p* = .015) to negative_LessSerious_ through willingness_LessSerious_, where with each simultaneous one‐unit increase in the predictors and willingness, participants were respectively .97, .95, and .94 times less likely to move toward intervening negatively. There were no significant indirect contributions found for more serious violence. Step 2 revealed that there were no indirect contributions on the less serious or more serious violence outcomes when perceived behavioural control and self‐efficacy were added.

#### Hypothesis 4

For significant interactions, simple slopes analyses (Aiken & West, 1991) were conducted to assess the contributions of the predictors at three levels of the moderators (1 *SD* below the mean, the mean, and 1 *SD* above the mean). Regarding social influences, the only significant interactions found were between positive attitudes, and both willingness measures (see Table 6), where positive attitudes more strongly predicted willingness_LessSerious_ and willingness_MoreSerious_ as subjective norms increased. With regards to control perceptions, no significant interactions were found.

**TABLE 6:** Standardized (β) estimates and (*p*‐values) for three‐level moderator effects predicting both willingness measures

	Willingness_LessSerious_			Willingness_MoreSerious_		
	Low	Med.	High	Low	Med.	High
Subjective norms × Positive attitudes	.07 (.057)	.14 (.000)	.21 (.000)	.07 (.050)	.13 (.000)	.19 (.000)

## DISCUSSION

This study is the first to assess the predictive ability of an augmented PWM (Gibbons & Gerrard, 1995, 1997) in an adolescent bystander intervention context. This was achieved using a prospective correlational design focusing on a complex interpersonal situation for young people, observing gender‐based violence. The results support the applicability of the augmented PWM in explaining how adolescents respond to their peers engaging in gender‐based violence. Overall, the results provide weak support for the integration of bidimensional attitudes, control perceptions in the form of perceived behavioural control, and moderators of social influences on attitudes into the PWM. There is, however, strong support for integrating control perceptions in the form of self‐efficacy into the model. Additionally, issues such as social influences appear to be an important first step when seeking to understand bystander intervention. Furthermore, findings partly support willingness as a predictor of intervention but not intentions, potentially a result of other influential factors.

Overall, the results in relation to Hypotheses 1a and 1b imply that bystander intervention can be governed by both reactive and deliberative decision‐making processes, but support for this was only found at the intentions and willingness levels of the decision‐making process, with more support being found for reactive decision‐making. This was highlighted by the stronger associations found for the predictors on willingness to intervene in more and less serious gender‐based violence. Furthermore, prototype perceptions, positive attitudes, and self‐efficacy were the largest predictors. As prototype perceptions have not been examined in the context of bystander decision‐making, this study has identified another key influence on bystanders considering intervention. That positive attitudes were a stronger predictor than negative attitudes overall aligns with other findings outside the bystander literature (Elliott et al., 2015; McCartan et al., 2018). The positive outcomes could therefore have greater urgency, making them more likely to influence decisions (Boucher & Osgood, 1969; Cacioppo et al., 1997). That self‐efficacy was one of the largest predictors aligns with other research highlighting its importance in bystander intervention (e.g. Banyard, 2008; McMahon et al., 2015; Sjögren et al., 2020; Sundstrom et al., 2018). This suggests that the addition of control perceptions in the form of self‐efficacy is an important addition to the PWM when considering bystander decision‐making.

However, at the behaviour level, things become less clear. Reactive decision‐making seems to impact positive and negative intervention behaviour in less serious violence but only when control perceptions were excluded from the model. Whereas there was no evidence for deliberative decision‐making from intentions found. Nevertheless, evidence for the predictive ability of the reactive decision‐making pathway is novel to the bystander literature, where intervention appears to be a dynamic process requiring reaction more so than a planned action. More generally, that reactive decision‐making better explains behaviour is consistent with findings in other contexts (Elliott et al., 2017; Gibbons et al., 1998, 2004; Rivis et al., 2006, 2010).

The null contributions of intentions, and willingness to intervene in more serious violence warrant discussion. Intentions predict behaviour in other bystander research (DeSmet et al., 2016; Leone & Parrott, 2019; McMahon et al., 2015; Rosval, 2013). Nevertheless, studies have also found no contributions (Austin et al., 2016; Báez et al., 2016; Borsky, 2015). Furthermore, contrasting findings on the predictive ability of intentions are apparent in other contexts (Orbell & Sheeran, 1998; Sheeran, 2002; Webb & Sheeran, 2006), suggesting a wider issue of translating intentions to behaviour (Gollwitzer, 1990; Gollwitzer & Sheeran, 2006). Indeed, more recent decision‐making models postulate that intentions only predict behaviour in facilitating environments where sufficient skills are possessed (Fishbein & Ajzen, 2010). Alternatively, intentions may be operating at a more abstract level. Given that they are measured by how likely it is that bystanders would intervene rather than referring to concrete plans (*I plan to intervene*) (see also Armitage et al., 2015, and Warshaw & Davis., 1985) in the current study, it could be that they are also tapping into other factors that may influence intervention more (Austin et al., 2016). As the predictive ability of willingness to intervene in more serious gender‐based violence has not been examined, it is difficult to draw concrete conclusions regarding this null result. Nevertheless, more serious violence is easily identifiable as “wrong” (Avery‐Leaf & Cascardi, 2004; Katz et al., 2011). Therefore, participants may overestimate their motivation to intervene but act contrarily when faced with the situation (Katz et al., 2011).

In support of Hypothesis 2, self‐efficacy predicted negative intervention in more serious gender‐based violence, in line with other research (McMahon et al., 2015; Sjögren et al., 2020). Self‐efficacy was the only predictor of negative intervention in more serious violence. Low beliefs in one's own capabilities to effectively intervene can therefore result in bystanders doing nothing or even laughing along. Negative intervention is encouraging to the perpetrator and can increase their status, leading them to perpetrate again (Salmivalli, 2014).

Disconfirming Hypothesis 2, no other predictions were found for control perceptions. Unexpectedly, perceived behavioural control positively predicted negative intervention in more serious violence, though the contribution was small. Participants who perceive more control over intervening positively may perceive more control over intervening negatively too or may simply choose to “do nothing.” However, when measuring control by the level of difficulty, Hoxmeier et al. (2018) found that those who intervened positively reported intervention to be easier. There appears to be an incongruency between the predictive abilities of controllability and perceived difficulty (Sparks et al., 1997). As this study is the first to measure controllability independently in the context of bystander intervention, more research is needed to disentangle its potential contributions.

In support of Hypothesis 3, mediation analyses showed some significant indirect contributions from positive attitudes, subjective norms, and prototype perceptions to both positive and negative intervention through the willingness to intervene in less serious violence. No significant indirect contributions were found through intentions, or willingness to intervene in more serious violence. This is likely due to intentions and willingness not directly predicting intervention in more serious violence, and the idea that participants may overestimate their willingness and intentions to intervene because of the ease of identifying “more serious” violence as wrong (e.g., Katz et al., 2011). Nevertheless, the significant findings are consistent with research demonstrating mediating contributions of willingness on predictor‐outcome relationships in different contexts (Elliott et al., 2017). Overall, the findings suggest that attitudes, subjective norms, and prototype perceptions are important antecedents to willingness to intervene in less serious gender‐based violence, and subsequently to positive and negative intervention in the decision‐making process.

In support of Hypothesis 4, some moderator contributions were found for subjective norms on positive attitudes. However, none were found for social influences on attitudes. Furthermore, none were found for attitudes on self‐efficacy or perceived behavioural control. Nevertheless, the significant findings align with the contingent consistency hypothesis (Acock & DeFleur, 1972), where attitudes are more predictive of behaviour when the social environment facilitates it. This study extends the applicability of the contingent‐consistency hypothesis to the PWM. There is therefore the need to assess intervention in a non‐additive fashion when considering the impact of other bystanders’ behaviours on positive attitudes. At the same time, the null findings suggest that there is also validity in examining the PWM additively. It seems that the contributions of attitudes are not impacted by comparison to the typical bystander (prototype perceptions). Similarly, the contributions of perceived behavioural control and self‐efficacy are not affected by how positive or negative a behaviour is evaluated.

### Strengths and weaknesses

Strengths of this study include the use of a prospective correlational design, the augmentation of a theoretical approach examining both reactive and deliberative decision‐making, and the inclusion of a large sample.

Another strength of this study is controlling for external social influences in the decision‐making process, highlighting the contributions of individual influences alone. One way this was achieved was by examining willingness by presenting participants with three hypothetical situations (no one else intervening, no one else there, and no friends intervening) impacting on bystander intervention (Latané & Darley, 1968; Levine et al., 2002; Oldenburg et al., 2018; Palmer & Abbott, 2018). Examining willingness to intervene despite these social influences gives a better insight into these individual factors. Furthermore, the three hypothetical situations were selected based on their similar impacts on intervention (e.g., Latané & Darley, 1968). However, it should be noted that the predictive differences between these situations were not explicitly tested in this study.

Although using a prospective correlational design was an advantage, it still limits our ability to infer causality in the relationships studied. Despite this, research examining social cognitive models often uses true correlational designs (Dohnke et al., 2015; Elliott et al., 2017; Rosval, 2013; Tappe, 2008; Thornberg & Jungert, 2014), and the prospective correlational design used here at least allows reports of behaviour to be temporally distanced from other variables assessed. Nonetheless, it would not be accurate to conclude, for example, that positive attitudes cause willingness to intervene when the reverse might be true. However, there are longitudinal studies evidencing causal relationships within the PWM (Armenta et al., 2015; Armitage & Talibudeen, 2010; Lewis et al., 2018; Litt & Lewis, 2016; O’Hara, 2012).

The number of significance tests that were conducted may raise concerns about an increased Type I error rate. While a Bonferroni correction can reduce this, it simultaneously increases the risk of a Type II error (Tabachnick & Fidell, 2007). Our study aimed to provide important insight into factors that influence bystander decision‐making. We therefore deemed it more important to not miss a possible effect. However, we have reported exact *p*‐values to allow others to interpret results according to different thresholds, should they wish.

The further analytical steps that were taken during the factor analyses may raise some concerns about the quality and the reliability of the measures used. Indeed, issues with reverse‐scored items and scales with fewer items did emerge. However, the steps taken to resolve these issues were driven by sound theoretical and empirical work (e.g., Ajzen, 2002; Little et al., 1999; Tomas & Oliver, 1999). Subsequently, the factor analyses elicited results showing that the final models all had an adequate or very good fit to the data (MacCallum et al., 1996). Furthermore, we endeavoured to also include Omega estimates within Table 1 to indicate each measure's internal consistency.

The overall contributions for the four intervention behaviours were small. This may be because other factors impact on intervention such as social influences (group membership with the victim/perpetrator: Levine et al., 2002; Levine & Crowther, 2008; Palmer & Abbott, 2018; Rutland & Killen, 2017; social status: Salmivalli, 2010, 2014). Ecological (campus size and cultural values: Banyard, 2008; school climate: Rosval, 2013) could have also influenced the current results. Including the examination of school‐level differences would have allowed for consideration of these ecological influences. Age was also an important predictor of nearly all outcomes, except negative intervention in more serious violence. Given that adolescence is a period in which there is much developmental and relationship change, the effects observed for age give an important insight into differences in bystander intervention behaviour at different developmental periods, where younger children report more positive and less negative intervention behaviour than older children (Banyard & Moynihan, 2011; Moynihan et al., 2015). Furthermore, studies have highlighted that intentions to intervene can differ depending on the perceived severity of incidents (Palmer et al., 2015). Though this was not measured in relation to different types of violence, it would be beneficial for future research to examine individuals’ perceptions of different types of violence to understand how this may affect bystander decision‐making.

Overall, this study aimed to examine individual factors that tend to be targeted through bystander intervention programmes. It is ascertained that intervention is nuanced (Wójcik & Mondry, 2020), and involves an interplay of individual, structural, and contextual factors, the examination of which was beyond the remit of this study.

## CONCLUSIONS

This study applied a novel approach to the examination of bystander intervention by testing an augmented Prototype Willingness Model to better understand individual influences on decision‐making. Overall, this study provides a dual process insight by examining both reactive and deliberative processes of individual decision‐making that can be challenged and changed through reduction efforts. Young people's intervention tends to be more reactive than deliberative, but only in less serious gender‐based violence contexts. Contrarily, in more serious gender‐based violence contexts, self‐efficacy was the key determinant of negative intervention such as doing nothing. Augmenting the PWM to include control perceptions in the form of self‐efficacy and bidimensional attitudes in the form of positive attitudes provides a better explanation of bystander decision‐making. However, the mixed moderator effects further highlight the complexity of this decision‐making process. Subjective norms and prototype perceptions are important social influences, where self‐comparison to the typical bystander was the largest influence of intentions and willingness. The findings of this study also have broader implications: the number of effects observed highlights the potential of utilizing the PWM to develop violence reduction strategies in schools. Furthermore, the age effects observed illustrate the importance of intervention at the earliest stages of high school settings.

## CONFLICT OF INTEREST

To the authors' best knowledge, there are no conflicts of interest.

## AUTHOR CONTRIBUTIONS

All authors have made substantial contributions to this work.

### Open Research Badges

All materials and data are publicly accessible via the Open Science Framework at [https://doi.org/10.17605/OSF.IO/C6GX7, https://doi.org/10.17605/OSF.IO/C6GX7, https://doi.org/10.17605/OSF.IO/C6GX7].

## Data Availability

The data that support the findings of this study are openly available on the Open Science Framework (OSF) at http://doi.org/10.17605/OSF.IO/C6GX7, reference number: osf.io/c6gx7.

## Footnotes

^1^
This study was part of a programme of three studies aiming to evaluate the effectiveness of a programme targeting gender‐based violence through bystander intervention. This age group was therefore determined by those participants who were eligible to receive the programme.
