# Supplementary material for: Bystander intervention among secondary school pupils: Testing an augmented Prototype Willingness Model
Source: Br J Soc Psychol. 2022 Mar 23;61(4):1221–44. doi: 10.1111/bjso.12534 (PMC9790461; doi:10.1111/bjso.12534)
Supplement: Supplementary file 1 [file BJSO-61-1221-s001.docx]

**Appendices**

**Appendix 1**

**Table S1.**

***Correlations between covariates and predictors***

|  | Age | Gender | Ethnicity | Affective empathy | Moral disengagement |
| --- | --- | --- | --- | --- | --- |
| Positive attitudes | .04 | .08* | -.03 | .18*** | -.12** |
| Negative attitudes | -.04 | .01 | .00 | -.01 | -.00 |
| Subjective norms | -.11* | .06* | -.01 | .17*** | -.19*** |
| Prototype perceptions | -.00 | .09* | -.00 | .20*** | -.23*** |
| PBC | -.03 | .02 | .01 | .14*** | -.21*** |
| Self-efficacy | -.01 | .02 | .05 | .19*** | -.25*** |
| Intentions | .05 | .20*** | -.02 | .18*** | -.09* |
| Willingness_LessSerious_ | -.08* | .17*** | -.01 | .35*** | -.31*** |
| Willingness_MoreSerious_ | .04 | .08 | -.03 | .26** | -.22** |

*Note.* * *p* < .05, ** *p* < .01, *** *p* < .001

**Appendix 2**

Positive items were combined and negative items were combined (see Table S1) in line with previous literature (Kärnä et al., 2010; Miller et al., 2012; Nocentini et al., 2013; Salmivalli et al., 1996, 2011). The literature highlights that positive intervention behaviour can include a variety of different behaviours including defending the victim as well as antegrade efforts such as reporting the violence to a teacher. Negative bystander behaviour can include joining in or reinforcing the violence by laughing along. It can also include passive behaviour, that is, not doing or saying anything, which can essentially also reinforce the violence.

**Table S2.**

***Positive and negative intervention possibilities from Miller et al. (2012)***

| **Positive or negative** | **Intervention possibility** |
| --- | --- |
| **Positive** | I told the person in public that acting like that was not ok |
|  | I told the person in private that acting like that was not ok |
|  | I talked to a member of staff/mentor about it privately |
|  | I talked to another adult |
| **Negative** | I didn’t do or say anything |
|  | I laughed or went along with it |

**Appendix 3**

*Tables containing factor score weights and factor loadings for each measure*

**Table S3.**

*Factor weights and loadings for two factor analyses on positive attitudes*

| **Positive attitudes** | **Factor weights**  **(factor loadings)** | |
| --- | --- | --- |
|  | FA1 | FA2 |
| **1a)** How positive would it be if you did something about it when you saw… A male peer / friend making rude or disrespectful comments about a girl’s body, clothes, or makeup? | .023 (.600) | .011  (.617) |
| **1b)** How positive would it be if you did something about it when you saw… A male peer / friend doing unwelcome or uninvited things toward a girl (or group of girls), such as howling, whistling, or making sexual gestures? | .026 (.606) | .019  (.636) |
| **2a)** How beneficial would it be if you did something about it when you saw… A male peer / friend spreading rumours about a girl's sexual reputation, like saying she's 'easy to get with’? | .054 (.740) | .028  (.761) |
| **2b)** How beneficial would it be if you did something about it when you saw… A male peer / friend arguing with a girl where he’s starting to swear at or threaten her? | .044 (.728) | .036  (.700) |
| **3a)** How rewarding would it be if you did something about it when you saw… A male peer / friend telling sexual jokes that disrespect women and girls? | .067 (.804) | .049  (.831) |
| **3b)** How rewarding would it be if you did something about it when you saw… A male peer / friend shoving, grabbing, or otherwise physically hurting a girl? | .073 (.814) | .048  (.804) |
| **4a)** How pleasant would it be if you did something about it when you saw… A male peer / friend showing other people sexual messages or naked/sexual pictures of a girl on a mobile phone or the internet? | .093 (.858) | .048  (.868) |
| **4b)** How pleasant would it be if you did something about it when you saw… A male peer / friend arguing with a girl where he’s starting to swear at or threaten her? | .089 (.855) | .072  (.882) |
| **5a)** How enjoyable would it be if you did something about it when you saw… A male peer / friend telling sexual jokes that disrespect women and girls? | .045 (.748) | .037  (.799) |
| **5b)** How enjoyable would it be if you did something about it when you saw… A male peer / friend taking sexual advantage of a girl (like touching, kissing, having sex with) who is drunk or high from drugs? | .040  (.748) | .031  (.796) |
| **6a)** How advantageous would it be if you did something about it when you saw… A male peer / friend showing other people sexual messages or naked/sexual pictures of a girl on a mobile phone or the internet? | .073  (.797) | .024  (.771) |
| **6b)** How advantageous would it be if you did something about it when you saw… A male peer / friend taking sexual advantage of a girl (like touching, kissing, having sex with) who is drunk or high from drugs? | .040  (.781) | .031 (.764) |

*Note.* FA1 = factor analysis on first half of dataset; FA2 = factor analysis on second half of dataset

**Table S4.**

*Factor weights and loadings for two factor analyses on negative attitudes*

| **Negative attitudes** | **Factor weights**  **(factor loadings)** | |
| --- | --- | --- |
|  | FA1 | FA2 |
| **1a)** How negative would it be if you did something about it when you saw… A male peer / friend making rude or disrespectful comments about a girl’s body, clothes, or makeup? | .024 (.763) | .025 (.686) |
| **1b)** How negative would it be if you did something about it when you saw… A male peer / friend arguing with a girl where he’s starting to swear at or threaten her? | .052 (.790) | .074 (.724) |
| **2a)** How harmful would it be if you did something about it when you saw… A male peer / friend showing other people sexual messages or naked/sexual pictures of a girl on a mobile phone or the internet? | .053 (.796) | .092 (.798) |
| **2b)** How harmful would it be if you did something about it when you saw… A male peer / friend shoving, grabbing, or otherwise physically hurting a girl? | .026 (.635) | .071 (.785) |
| **3a)** How unrewarding would it be if you did something about it when you saw… A male peer / friend telling sexual jokes that disrespect women and girls? | .029 (.747) | .071 (.772) |
| **3b)** How unrewarding would it be if you did something about it when you saw… A male peer / friend doing unwelcome or uninvited things toward a girl (or group of girls), such as howling, whistling, or making sexual gestures? | .038 (.759) | .070 (.773) |
| **4a)** How unpleasant would it be if you did something about it when you saw… A male peer / friend making rude or disrespectful comments about a girl’s body, clothing, or makeup? | .045 (.831) | .078 (.800) |
| **4b)** How unpleasant would it be if you did something about it when you saw… A male peer / friend arguing with a girl where he’s starting to swear at or threaten her? | .057 (.838) | .081 (.803) |
| **5a)** How unenjoyable would it be if you did something about it when you saw… A male peer / friend showing other people sexual messages or naked/sexual pictures of a girl on a mobile phone or the internet? | .017 (.634) | .030 (.596) |
| **5b)** How unenjoyable would it be if you did something about it when you saw… A male peer / friend shoving, grabbing, or otherwise physically hurting a girl? | .020 (.641) | .028 (.596) |
| **6a)** How disadvantageous would it be if you did something about it when you saw… A male peer / friend spreading rumours about a girl's sexual reputation, like saying she's 'easy to get with’? | .054 (.808) | .092 (.809) |
| **6b)** How disadvantageous would it be if you did something about it when you saw… A male peer / friend taking sexual advantage of a girl (like touching, kissing, having sex with) who is drunk or high from drugs? | .042 (799) | .090 (.812) |

*Note.* FA1 = factor analysis on first half of dataset; FA2 = factor analysis on second half of dataset

**Table S5.**

*Factor weights and loadings for two factor analyses on self-efficacy and perceived behavioural control*

|  | | Factor weights  (factor loadings) | |
| --- | --- | --- | --- |
|  |  | **FA1** | **FA2** |
|  | **Self-efficacy** |  |  |
| Parcelled  items | **1a)** Over the next month, how confident are you that you will be able to do something about it when you see…A male peer / friend telling sexual jokes that disrespect women and girls? | .159  (.746) | .136  (.692) |
|  | **2a)** Over the next month, I have the ability to do something about it when I see…A male peer / friend spreading rumours about a girl's sexual reputation, like saying she's 'easy to get with’ |  |  |
| Parcelled items | **1b)** Over the next month, how confident are you that you will be able to do something about it when you see…A male peer / friend taking sexual advantage of a girl (like touching, kissing, having sex with) who is drunk or high from drugs? | .101  (.703) | .113  (.686) |
|  | **2b)** Over the next month, I have the ability to do something about it when I see… A male peer / friend shoving, grabbing, or otherwise physically hurting a girl |  |  |
| Individual  items | **3a)** Over the next month, to what extent do you see yourself as being capable of doing something about it when you see… A male peer / friend making rude or disrespectful comments about a girl’s body, clothes, or makeup? | .052  (.522) | .043  (.471) |
|  | **3b)** Over the next month, to what extent do YOU see yourself as being capable of doing something about it when you see… A male peer / friend taking sexual advantage of a girl (like touching, kissing, having sex with) who is drunk or high from drugs? | .017  (.471) | .037  (.471) |
|  | **Perceived behavioural control** |  |  |
| Parcelled  items | **1a)** Over the next month, it is completely up to ME whether or not I do something about it when I see… A male peer / friend showing other people sexual messages or naked/sexual pictures of a girl on a mobile phone or the internet | .272  (.908) | .295  (.910) |
|  | **2a)** Over the next month, how much personal control do YOU feel you have over doing something about it when you see… A male peer / friend spreading rumours about a girl's sexual reputation, like saying she's 'easy to get with’? |  |  |
| Parcelled  items | **1b)** Over the next month, it is completely up to ME whether or not I do something about it when I see… A male peer / friend doing unwelcome or uninvited things toward a girl (or group of girls), such as howling, whistling, or making sexual gestures | .249  (.901) | 246  (.893) |
|  | **2b**) Over the next month, how much personal control do YOU feel you have over doing something about it when you see… A male peer / friend arguing with a girl where he’s starting to swear at or threaten her? |  |  |

*Note.* FA1 = factor analysis on first half of dataset; FA2 = factor analysis on second half of dataset

**Table S6.**

*Factor weights and loadings for two factor analyses on prototype perceptions*

| Prototype perceptions | Factor weights  (factor loadings) | |
| --- | --- | --- |
|  | FA1 | FA2 |
| 1a) Do you resemble the type of person your age that regularly does something about it when they see…A male peer / friend making rude or disrespectful comments about a girl’s body, clothes, or makeup? | .140  (.756) | .129  (.762) |
| 1b) Do you resemble the type of person your age that regularly does something about it when they see…A male peer / friend arguing with a girl where he’s starting to swear at or threaten her? | .146  (.762) | .090  (.744) |
| 2a) How similar or different are you to the type of person your age that regularly does something about it when they see…A male peer / friend spreading rumours about a girl's sexual reputation, like saying she's 'easy to get with’? | .169  (.769) | .155  (.771) |
| 2b) How similar or different are you to the type of person your age that regularly does something about it when they see…A male peer / friend taking sexual advantage of a girl (like touching, kissing, having sex with) who is drunk or high from drugs? | .113  (.748) | .069  (.718) |
| 4a) To what extent are you like the type of person your age that regularly does something about it when they see…A male peer / friend showing other people sexual messages or naked/sexual pictures of a girl on a mobile phone or the internet? | .140  (.736) | .174  (.833) |
| 4b) To what extent are you like the type of person your age that regularly does something about it when they see…A male peer / friend doing unwelcome or uninvited things toward a girl (or group of girls), such as howling, whistling, or making sexual gestures? | 103  (.720) | .214  (.839) |
| Deleted items |  |  |
| 3a) I am comparable to the type of person my age that regularly does something about it when they see… A male peer / friend telling sexual jokes that disrespect women and girls |  |  |
| 3b) I am comparable to the type of person my age that regularly does something about it when they see… A male peer / friend shoving, grabbing, or otherwise physically hurting a girl |  |  |

*Note.* FA1 = factor analysis on first half of dataset; FA2 = factor analysis on second half of dataset

**Table S7.**

*Factor weights and loadings for two factor analyses on subjective norms*

| Subjective norms | Factor weights  (factor loadings) | |
| --- | --- | --- |
|  | FA1 | FA2 |
| 2a) Of the students you know, how many do you think will do something about it over the next month when they see… A male peer / friend spreading rumours about a girl's sexual reputation, like saying she's 'easy to get with’? | .252  (.838) | .201  (.815) |
| 2b) Of the students you know, how many do you think will do something about it over the next month when they see… A male peer / friend shoving, grabbing, or otherwise physically hurting a girl? | .107  (.689) | .128  (.747) |
| 3a) Over the next month, how often do you think that other students will do something about it when they see…A male peer / friend showing other people sexual messages or naked/sexual pictures of a girl on a mobile phone or the internet? | .070  (.644) | .079  (.687) |
| 3b) Over the next month, how often do you think that other students will do something about it when they see… A male peer / friend doing unwelcome or uninvited things toward a girl (or group of girls), such as howling, whistling, or making sexual gestures? | .039  (.582) | .048  (.627) |
| Deleted items |  |  |
| 1a) Over the next month, do you think many students would do something about it if they saw… A male peer / friend making rude or disrespectful comments about a girl’s body, clothes, or makeup? |  |  |
| 1b) Over the next month, do you think many students would do something about it if they saw… A male peer / friend arguing with a girl where he’s starting to swear at or threaten her? |  |  |

*Note.* FA1 = factor analysis on first half of dataset; FA2 = factor analysis on second half of dataset

**Table S8.**

*Factor weights and loadings for two factor analyses on intentions*

| **Intentions** | **Factor weights**  **(factor loadings)** | |
| --- | --- | --- |
|  | FA1 | FA2 |
| **1.** How likely (are you) to do something about it over the next month if a male peer / friend is…Making rude or disrespectful comments about a girl’s body, clothes, or makeup | .125  (.833) | -.038  (.707) |
| **2.** How likely (are you) to do something about it over the next month if a male peer / friend is…Telling sexual jokes that disrespect women and girls | .120  (.860) | .045  (.786) |
| **3.** How likely (are you) to do something about it over the next month if a male peer / friend is…Spreading rumours about a girl's sexual reputation, like saying she's 'easy to get with’ | .136  (.880) | .232  (.895) |
| **4.** How likely (are you) to do something about it over the next month if a male peer / friend is…Showing other people sexual messages or naked/sexual pictures of a girl on a mobile phone or the internet | .177  (.880) | .281  (.914) |
| **5.** How likely (are you) to do something about it over the next month if a male peer / friend is…Doing unwelcome or uninvited things toward a girl (or group of girls), such as howling, whistling, or making sexual gestures | .109  (.865) | .118  (.834) |
| **6.** How likely (are you) to do something about it over the next month if a male peer / friend is…Arguing with a girl where he’s starting to swear at or threaten her | .069  (.848) | -.012  (.741) |
| **7.** How likely (are you) to do something about it over the next month if a male peer / friend is…Shoving, grabbing, or otherwise physically hurting a girl | .046  (.851) | .007  (.774) |
| **8.** How likely (are you) to do something about it over the next month if a male peer / friend is…Taking sexual advantage of a girl (like touching, kissing, having sex with) who is drunk or high from drugs | .043  (.815) | .106  (.835) |

*Note.* FA1 = factor analysis on first half of dataset; FA2 = factor analysis on second half of dataset

**Table S9.**

*Factor weights and loadings for two factor analyses on both willingness measures*

|  | Factor weights  (factor loadings) | |
| --- | --- | --- |
|  | **FA1** | **FA2** |
| Willingness_LessSerious_ |  |  |
| 1a) Over the next month, suppose you saw…A male peer / friend making rude or disrespectful comments about a girl’s body, clothes, or makeup, and no-one else there was doing anything about it. How willing would you be to do something about it? | .101  (.769) | .088  (.760) |
| 2a) Over the next month, would you be willing to do something about it if you saw…A male peer / friend telling sexual jokes that make fun of women and girls and none of your friends there were doing anything about it? | .197  (.892) | .196  (.905) |
| 2b) Over the next month, would you be willing to do something about it if you saw… A male peer / friend arguing with a girl where he’s starting to swear at or threaten her and none of your friends there were doing anything about it? | .128  (.421) | .143  (.445) |
| 3a) Imagine that there were no other students around. To what extent would you be willing to do something about it over the next month when you see…A male peer / friend showing other people sexual messages or naked/sexual pictures of a girl on a mobile phone or the internet? | .066  (.745) | .065  (.753) |
| Willingness_MoreSerious_ |  |  |
| 1b) Over the next month, suppose you saw…A male peer / friend shoving, grabbing, or otherwise physically hurting a girl and no-one else there was doing anything about it. How willing would you be to do something about it? | .169  (.809) | .112  (.763) |
| 2b) Over the next month, would you be willing to do something about it if you saw… A male peer / friend arguing with a girl where he’s starting to swear at or threaten her and none of your friends there were doing anything about it? | .156  (.499) | .171  (.495) |
| 3b) Imagine that there were no other students around. To what extent would you be willing to do something about it over the next month when you see…A male peer / friend shoving, grabbing, or otherwise physically hurting a girl? | .144  (.816) | .183  (.843) |

*Note.* FA1 = factor analysis on first half of dataset; FA2 = factor analysis on second half of dataset

**Appendix 4**

*Workings to determine whether R^2^* *change scores were significant between steps 1 and 2*

The below equation was employed from Tabachnick & Fidell (1996) to calculate the *F* increment score in order to understand if the *R*^2^ scores significantly increased when perceived behavioural control and self-efficacy were added to the model.

**Figure S1.**

*Equation used to calculate F incremental score.*

*
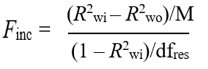
*

*Note. F*_inc_ = F incremental score; *R*^2^_wi_ = *R*^2^ coefficient at step 2 (with the addition of perceived behavioural control and self-efficacy); *R*^2^_wo_ = *R*^2^ coefficient at step 1 (without the addition of perceived behavioural control and self-efficacy); M = the number of IVs added (in this case there are two, perceived behavioural control and self-efficacy); df_res_ = N (total number of cases) – k (total number of IVs) – 1.

**Figure S2.**

*Workings for each of the outcomes to determine if R^2^ change is significant.*

| **Intentions:**  *F_inc_ =* (0.224 – 0.198)/2    (1 – 0.224)/1029  *F_inc_ =* 17.24, df = 4, 6  Increase is significant at p < .01 | **Willingness_LessSerious_:**  *F_inc_ =* (0.440 – 0.351)/2    (1 – 0.440)/1029  *F_inc_ =* 81.76, df = 4, 6  Increase is significant at p < .001 |
| --- | --- |
| **Negative_LessSerious_:**  *F_inc_ =* (0.035 – 0.032)/2    (1 – 0.035)/1026  *F_inc_ =* 3.19 , df = 8, 10  Increase is significant at p < .05 | **Positive_LessSerious_:**  *F_inc_ =* (0.049 – 0.044)/2    (1 – 0.049)/1026  *F_inc_ =* 5.39 , df = 8, 10  Increase is significant at p < .01 |
| **Willingness_MoreSerious_:**  *F_inc_ =* (0.371 – 0.305)/2    (1 – 0.371)/704  *F_inc_ =* 36.93 , df = 4, 6  Increase is significant at p < .001 | **Positive_MoreSerious_:**  *F_inc_ =* (0.058 – 0.051)/2    (1 – 0.058)/700  *F_inc_ =* 2.60 , df = 8, 10  Increase is not significant |
| **Negative_MoreSerious_:**  *F_inc_ =* (0.061 – 0.042)/2    (1 – 0.061)/700  *F_inc_ =* 7.08, df = 8, 10  Increase is significant at p < .01 |  |

References:

Kärnä, A., Voeten, M., Poskiparta, E., & Salmivalli, C. (2010). Vulnerable children in varying classroom contexts: Bystanders’

behaviors moderate the effects of risk factors on victimization. *Merrill-Palmer Quarterly*, 56(3), 261–282.

https://doi.org/10.1353/mpq.0.0052

Nocentini, A., Menesini, E., & Salmivalli, C. (2013). Level and change of bullying behaviour during high school: A multilevel growth

curve analysis. *Journal of Adolescence*, 36(3), 495–505. <https://doi.org/10.1016/j.adolescence.2013.02.004>

Salmivalli, C., Lagerspetz, K., Björkqvist, K., Ostermalm, K., & Kaukiainen, A. (1996). Bullying as a group process: Participant

roles and their relations to social status within the group. *Aggressive Behaviour*, 22(8), 1–15.

Salmivalli, C., Voeten, M., & Poskiparta, E. (2011). Bystanders matter: Associations between reinforcing, defending, and the

frequency of bullying behavior in classrooms. *Journal of Clinical Child & Adolescent Psychology*, 40(5), 668–676.

https://doi.org/10.1080/15374416.2011.597090
